# Association of Single-Nucleotide Polymorphisms in Sweet Taste Perception and Intake Genes with Primary Ciliary Dyskinesia and Its Clinical Phenotypes

**DOI:** 10.3390/ijms27031234

**Published:** 2026-01-26

**Authors:** Gioia Piatti, Mirko Aldè, Romina Ruberto, Aurora Santin, Giorgia Girotto, Maria Pina Concas

**Affiliations:** 1 Department of Pathophysiology and Transplantation, University of Milan, 20122 Milan, Italy; 2 Department of Specialist Surgical Sciences, Fondazione IRCCS Ca’ Granda Ospedale Maggiore Policlinico, 20122 Milan, Italy; mirko.alde@unimi.it; 3 Institute for Maternal and Child Health, IRCCS “Burlo Garofolo”, 34137 Trieste, Italy; romina.ruberto@burlo.trieste.it (R.R.); giorgia.girotto@burlo.trieste.it (G.G.); mariapina.concas@burlo.trieste.it (M.P.C.); 4 Department of Medicine, Surgery and Health Sciences, University of Trieste, 34149 Trieste, Italy; aurora.santin@burlo.trieste.it

**Keywords:** primary ciliary dyskinesia, extra oral taste receptors, sweet taste signaling, immune regulation, respiratory infections, *SLC2A4*, *FGF21*

## Abstract

Primary ciliary dyskinesia (PCD) is a congenital motile ciliopathy causing impaired mucociliary clearance and characterized by recurrent respiratory infections affecting both the upper and lower airways. Several genes involved in taste perception pathways are expressed in extraoral tissues and have recently emerged as regulators of airway immune responses. This study aimed to (1) analyze potential correlations between PCD clinical manifestations and (2) investigate whether genetic variants within sweet signaling genes (SweetG) could be associated with PCD features. A total of 17 SNPs in nine SweetG were tested for differences in allele frequency between patients and the gnomAD European reference population using a binomial test. Regression models were used to evaluate associations between SweetG-SNPs and clinical features of patients. A cohort of 34 patients (10–69 years, 44.1% male) was included in the study. Regarding (1), a moderate/high correlation was identified among the clinical manifestations of the pathologies. Regarding (2), the minor alleles of rs5415 (*SLC2A4* gene) and rs838133 (*FGF21* gene) were less frequent in patients than in the reference population (*p* < 0.05). In addition, rs5415 and rs838133 were associated with the presence of chronic rhinosinusitis and *situs inversus*, respectively (*p* < 0.05). This study reveals associations between SweetG-SNPs and PCD as well as its specific clinical features, suggesting a potential link between sweet signaling pathways and PCD clinical variability. Although larger multicenter studies are warranted to validate these findings, they represent a promising area of research that can enhance our understanding of PCD and elucidate the genetic basis of clinical manifestations associated with the disease.

## 1. Introduction

Primary ciliary dyskinesia (PCD) is a rare, congenital motile ciliopathy (OMIM: 244400) caused by structural and/or functional defects in motile cilia. These microscopic, hair-like organelles line the respiratory tract and are essential for effective mucociliary clearance. Indeed, in patients with PCD, impaired ciliary motility leads to chronic retention of mucus and pathogens, thus resulting in recurrent and chronic infections of the upper and lower respiratory tracts [1]. Over time, recurrent infections and chronic inflammation can lead to chronic rhinosinusitis (CRS) and otitis media in the upper airways, while in the lower airways, they cause bronchitis and the development of bronchiectasis, typically affecting the lingula and middle lobe, as well as both lower lobes. Progressive lung damage leads to a decline in pulmonary function and may progress to respiratory failure [2,3]. Moreover, approximately 50% of patients present organ laterality defects, most commonly *situs inversus*, due to altered nodal cilia function during embryonic development [4].

Recent studies have highlighted intriguing connections between taste perception pathways and immune regulation in the respiratory system. In particular, genes associated with taste signaling, such as those encoding taste receptors and related signaling molecules, have been found to be expressed in extraoral tissues, including the airway epithelium [5,6]. Indeed, members of the bitter (T2Rs) and sweet (T1Rs) families, beyond modulating taste perception and intake, are reported to play a role in the modulation of innate immune responses in extra-oral body districts [7]; specifically, the activity of these receptors can modulate antimicrobial peptides secretion in the respiratory tract, thus suggesting a potential link between chemosensory signaling and immune defense mechanisms [8]. The modulation of the immune response to bacteria mediated by these receptors is particularly important in PCD, which is characterized by recurrent respiratory infections, as genetic variations in taste receptors may help explain individual differences in disease progression and microbiological outcomes. Specifically, activation of T1Rs negatively regulates T2R signaling, thus altering ciliary beat frequency and mucus production, hence modulating the balance between microbial detection and host defense [9]. To date, T2Rs and T1Rs have been deeply investigated for their role in activating innate immune responses, while other genes involved in sweet taste signaling have received less attention. For instance, glucose transporters, such as *SLC2A4* (*GLUT4*), are reported to modulate local glucose levels in airway epithelial cells, thus influencing microbial growth and inflammatory signaling [10]. Members of the *FGF* family, including *FGF21*, not only regulate sweet perception [11] but also influence developmental, immune, and inflammatory pathways [12]. Moreover, *FTO*, a key regulator of energy metabolism and body mass, also influences immune cell function [13]. Hence, genes belonging to the sweet signaling pathways (SweetG) could be considered mediators linking sweet taste signaling and airway immunity, providing a rationale for their investigation in PCD patients who experience chronic and recurrent respiratory infections.

Overall, these findings suggest that chemosensory and metabolic pathways involving sweet taste signaling and glucose transport could be relevant in airway diseases characterized by recurrent respiratory infections. Hence, this study aims to (1) analyze potential correlations between PCD clinical manifestations and (2) investigate whether genetic variants within sweet signaling genes could be associated with PCD features from the perspective of identifying novel genes that may contribute to the clinical heterogeneity of PCD.

## 2. Results

### 2.1. Sample Characteristics

The study comprised a cohort of 34 PCD patients (aged 10–69 years; 44.1% male). In Table 1, the clinical characteristics of the cohort are summarized. Diagnostic findings in patients with a definitive diagnosis of PCD are shown in Table S1.

### 2.2. Interrelationships Among Clinical Parameters in PCD Patients

**Figure 1:**
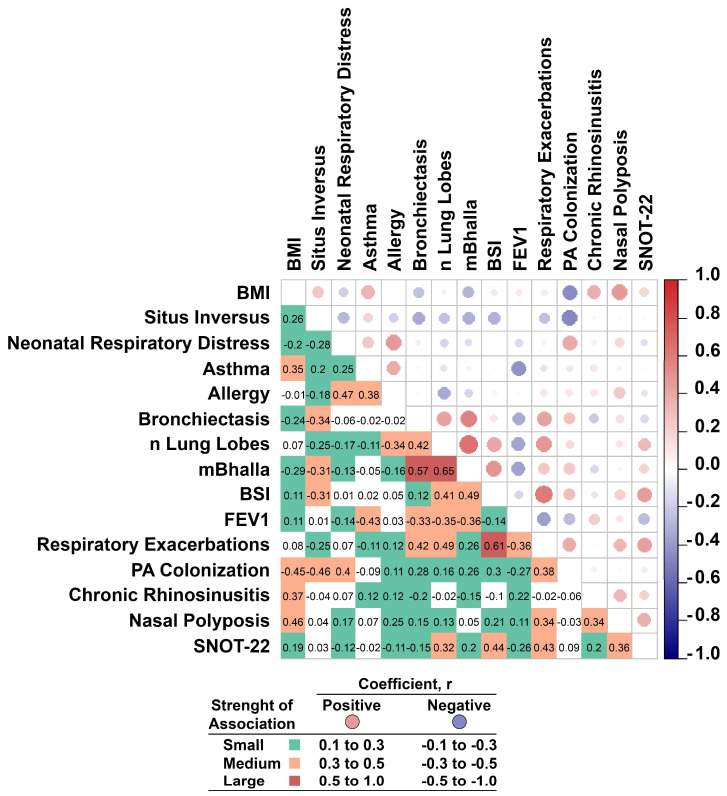
The matrix displays the strength and direction of correlations between PCD clinical variables. The correlation direction is represented by circular markers, whose size and intensity reflect the sign and magnitude of the coefficient. Correlation strength is categorized by cell color into three levels—small, medium, and large—as defined in the legend. Intervals are left-closed and right-open, except for the last, which includes ±1.0.

To deepen the clinical variability of PCD patients, a pairwise correlation matrix was employed (Figure 1). Among the results, Body Mass Index (BMI) was positively associated with nasal polyposis and CRS, suggesting a potential link between increased body mass and upper airway inflammation.

Neonatal respiratory distress correlated with both allergy and PA colonization, highlighting that respiratory vulnerability, which happens in early life, could be a possible factor influencing immune and infectious outcomes in adults.

Asthma and allergy showed a moderate positive correlation, with asthma also being negatively associated with FEV_1_, highlighting its impact on respiratory function. Moreover, lung parameters such as the extent of bronchiectasis, the number of affected lung lobes, and the Bhalla score were strongly interrelated. The Bronchiectasis Severity Index (BSI) correlated significantly with both respiratory exacerbations and sinonasal symptom burden (SNOT-22). Finally, SNOT-22 scores were positively linked to nasal polyposis and respiratory exacerbations, emphasizing the interplay between upper and lower airway involvement in PCD patients.

### 2.3. Allele Frequency Comparison Between PCD Patients and gnomAD Reference Population

All the selected SNPs (Table S2) were compared for differences in allele frequencies between PCD patients and the reference population. Statistically significant differences (*p* < 0.05, Table 2 and Table S3) were observed for four SNPs located in two genes: rs838133 and rs838145 within the *FGF21* gene and rs2654185 and rs5415 within the *SLC2A4* gene. In all cases, the major allele was consistently overrepresented in PCD patients compared with the reference population.

### 2.4. Associations Between the Most Significant SNPs and PCD Clinical Features

To explore potential genetic associations with PCD clinical features, the most significant variants identified in the previous analysis were tested for relationships with specific disease traits. All the results are provided in Table S4. Two variants showed statistically significant associations: (1) rs838133 within the *FGF21* gene was associated with *situs inversus* and (2) rs5415 within the *SLC2A4* gene was associated with CRS.

**Figure 2:**
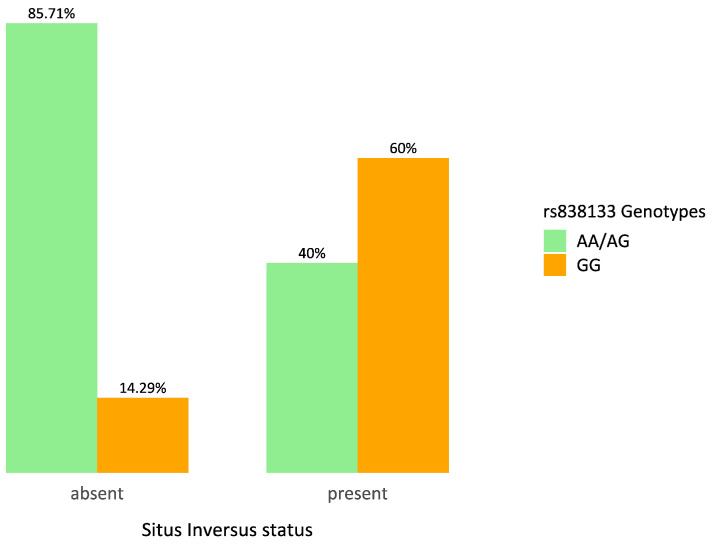
Distribution of rs838133 (*FGF21*) genotypes in relation to *situs inversus* status. The bar plot shows the relative frequency (%) of individuals carrying AA/AG versus GG genotypes across *situs inversus* status (absent/present).

Regarding (1), as shown in Figure 2, among individuals without *situs inversus*, only two subjects (14.3%) were homozygous for the alternative allele (GG), while the majority (85.7%) carried the reference allele (AA) or were heterozygous (AG) for rs838133 (*FGF21*). In contrast, among individuals with *situs inversus*, the percentage of GG reached 60.0% (12 subjects).

These genotype frequencies suggest an enrichment of the GG genotype among individuals affected by *situs inversus*. Statistical analysis using Fisher’s exact test confirmed the significance of this association (*p* = 0.0128).

**Figure 3:**
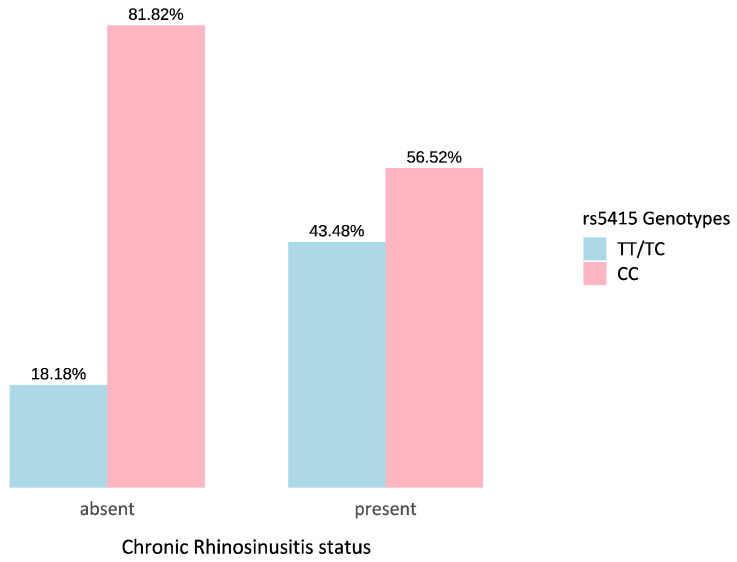
Distribution of rs5415 (*SLC2A4*) genotypes in relation to CRS status. The bar plot shows the relative frequency (%) of individuals carrying TT/TC versus CC genotypes across CRS status (absent/present).

Concerning (2), as shown in Figure 3, among individuals without CRS, only two subjects (18.2%) were homozygous for the reference allele T (genotypes TT or CT), while the majority (81.8%) were homozygous for the alternative allele C (genotype CC) of rs5415 (*SLC2A4*). In contrast, among individuals with CRS, the percentage of TT/CT carriers increased to 43.5% (10 subjects).

These genotype frequencies indicate a relative increase in the prevalence of the CC genotype among individuals without CRS. Logistic regression analysis suggests the protective effect of the CC genotype (OR = 0.0052, *p* = 0.0492).

## 3. Discussion

PCD is recognized as a rare congenital motile ciliopathy caused by pathogenic variants within genes encoding structural ciliary components; however, current evidence is pointing out that genetic modifiers outside the ciliary genes may shape the variability of its clinical presentations. In this light, genes involved in chemosensory pathways, such as those regulating sweet taste perception, are emerging as potential modulators of immunity and inflammatory processes [7,9,14].

We analyzed potential correlations among clinical manifestations of PCD and investigated whether genetic variants in sweet signaling genes are associated with PCD features. The correlation analysis highlighted a positive relationship between BMI, nasal polyposis, and CRS, in line with previous studies linking obesity and altered glucose metabolism to chronic inflammatory airway diseases [15].

Furthermore, a correlation between the sinonasal disease severity (SNOT-22), respiratory exacerbations, and bronchiectasis highlighted the functional continuum between upper and lower airways in PCD.

Concerning whether genetic variants in sweet signaling genes are associated with PCD features, in this cohort, four variants within two genes, *FGF21* and *SLC2A4*, displayed significant differences in allele frequency compared with the European reference population. Among these variants, we observed two statistically significant associations with PCD features: rs5415 (*SLC2A4/GLUT4*) with CRS and rs838133 (*FGF21*) with *situs inversus*.

The first significant finding regards the association between rs5415 within *SLC2A4* and CRS in PCD patients. *SLC2A4* encodes GLUT4, an insulin-regulated glucose transporter primarily expressed in muscle and adipose tissue but also detected in respiratory epithelial cells [16]. *SLC2A4* is best known for its role in insulin-regulated glucose uptake in muscle and adipose tissue, but it has also been linked to inflammatory signaling in CRS through regulation of NF-κB-dependent pathways [10]. In CRS, elevated glucose levels in nasal secretions have been reported, creating a nutrient-rich environment that fosters bacterial growth and sustains chronic inflammation [17,18]. Indeed, the airway surface liquid (ASL) normally contains a low glucose concentration (~0.4 mM), which restricts bacterial growth and supports mucosal defense [19,20]. Disruption of this homeostasis could allow the proliferation of pathogens, such as *Staphylococcus aureus* and *Pseudomonas aeruginosa,* and other *Gram-negative* bacteria [21,22] capable of using glucose as a carbon source, exacerbating airway inflammation. Clinically, this mechanism contributes to recurrent respiratory exacerbations and chronic disease progression, as observed in other disorders with impaired mucosal glucose regulation, such as cystic fibrosis and CRS [17]. In the context of PCD, where impaired mucociliary clearance can predispose to chronic infections, variation in *SLC2A4* may further modulate local glucose availability in the airway surface liquid, thus influencing microbial colonization and the intensity of inflammation. These findings suggest that genetic variation in glucose-handling pathways could be involved in modulating the sinonasal disease severity in PCD. Further studies and functional experiments will be required to confirm these associations and clarify the underlying molecular mechanisms.

Concerning *FGF21*, it encodes Fibroblast Growth Factor 21, known for its role in glucose and lipid metabolism. According to the current literature, members of the same family (FGFs) of *FGF21* have been implicated in left–right patterning and ciliary signaling [23,24]. Indeed, in classical PCD, laterality defects are typically explained by dysfunctional nodal cilia, which fail to generate the leftward flow required for asymmetry determination [25]. Although there is currently no direct evidence linking *FGF21* to motile ciliary function, it represents a novel candidate that may influence laterality determination indirectly, potentially through regulation of metabolic signaling, modulation of growth hormone pathways, or interactions with key morphogenetic pathways such as TGF-β, Wnt, or Hedgehog [26]. Further functional studies are certainly needed to deepen these results in order to understand whether *FGF21* could act as a genetic modifier influencing the laterality defects in PCD.

Overall, this is the first study that investigates variants within sweet taste signaling in PCD. While these SweetG could be considered promising candidates for modulating airway immunity, their role in PCD remains to be deepened and requires confirmation through future functional experiments and studies in larger cohorts.

However, this represents a promising area of research that may enhance our understanding of PCD and elucidate the genetic basis of respiratory infections associated with this disease. In particular, elucidating the genetic basis of the respiratory infections related to PCD could represent a significant step forward, also in pediatric settings, where early diagnosis and targeted genetic insights could greatly improve long-term outcomes for children with the disease.

In conclusion, this study provides novel insights into how chemosensory pathways may impact the clinical variability of PCD. Our results suggest that *SLC2A4* and *FGF21* genes may play a role in the disease, as variants in these genes were less prevalent in patients with PCD compared to the reference population. Moreover, SNPs in *SLC2A4* and *FGF21* were associated with specific PCD clinical features, such as chronic rhinosinusitis and *situs inversus*, respectively. Overall, these findings highlight significant associations between genes involved in sweet signaling pathways and PCD susceptibility and phenotype, opening novel insights into the complex genetic architecture underlying this disorder.

## 4. Materials and Methods

### 4.1. Clinical Data

Thirty-four patients with PCD, diagnosed and followed at the Unit of Respiratory Diseases, Policlinico Hospital, Milan, were enrolled in the study. A definitive diagnosis of PCD was based on clinical features together with evidence of ciliary ultrastructural defects, abnormal ciliary function, or the presence of a known pathogenic genetic mutation [27,28].

Clinical data were collected during routine follow-up visits, which typically occurred every three to four months. Patients were clinically stable and free from acute respiratory infections for at least 30 days prior to data collection.

Clinical history data were collected with a particular focus on the presence of recurrent upper and lower respiratory infections, as well as on the frequency and severity of exacerbations (categorized as <2 or ≥2 per year). Additional variables included Body Mass Index (BMI), allergic status, presence of bronchial asthma, chronic rhinosinusitis (CRS), history of sinonasal surgery, and presence of bronchiectasis.

CRS was diagnosed according to European guidelines [29] based on findings from nasal endoscopy or sinus computed tomography (CT). The most recent CT or Magnetic Resonance Imaging (MRI) scan available was used to calculate the Lund–Mackay score (LM). Each sinus group was graded on a scale from 0 to 2 (0 = no abnormality, 1 = partial opacification, 2 = total opacification). The ostiomeatal complex was scored as 0 (not obstructed) or 2 (obstructed). The total LM score ranges from 0 to 24, with each side of the sinus evaluated separately on a scale of 0 to 12 [30].

Patients completed the SNOT-22 questionnaire, a widely used instrument for assessing patient-reported outcomes in CRS. The questionnaire includes 22 items, each rated on a scale from 0 (no problem) to 5 (high impact on quality of life), covering rhinological, ear/facial, general, physical, and psychological domains. The scores range from 0 to 110, with higher scores indicating more severe symptoms [31].

Respiratory function was assessed by spirometry, performed according to the European Thoracic Society (ERS) guidelines [32]. The results were expressed as a percentage of the predicted values based on the patient’s height and age. Lung volumes and airflow rates were considered normal when greater than 80% of the expected value. Typically, each patient underwent at least one spirometry per year; for this study, the most recent available examination was considered.

The extent of bronchiectasis, severity of bronchial dilatation, bronchial wall thickness, presence of mucus plugging in large and small airways, and decreased parenchymal attenuation were evaluated on chest CT images for each lobe using the modified Bhalla high-resolution computed tomography scoring system (mBhalla) [33]. The lingula was considered a separate lobe. In cases where a lobectomy had been performed, a severity score of 3 was arbitrarily assigned to the missing lobe, and the distribution of abnormalities was assumed to be diffuse. For each abnormality, the mean score across all lobes was calculated, and lobar predominance was assessed. The total CT scores ranged from 0 to 48.

In addition to the mBhalla scoring system, the Bronchiectasis Severity Index (BSI) was also calculated, a multidimensional scoring system created and validated to classify the severity of bronchiectasis [34].

Microbiological data were obtained from all available sputum cultures. Each patient had at least three sputum bacteriology assessments per year, and only those with microbiological data spanning at least one year were included in the analysis. Chronic bronchial infection was defined as the isolation of the same pathogen from sputum culture on two or more occasions at least three months apart within a one-year period [35]. For *Pseudomonas aeruginosa* (PA) colonization, patients were classified as non-colonized if they had never tested positive or had only a single positive culture for PA. Patients with at least two positive sputum cultures for PA, three months apart within one year, were classified as colonized [2].

Exacerbations were defined according to the expert consensus criteria for PCD [36]. Considering that the median number of exacerbations in our patient cohort was two per year prior to analysis, we classified patients into two groups: low-EXAC: (<2 exacerbations/year) and high-EXAC (≥2 exacerbations/year).

### 4.2. Genotyping and Genetic Analysis

For each enrolled patient, a peripheral whole blood sample was collected for DNA extraction and genotyping. Genomic DNA was extracted using the QIAsymphony^®^ SP instrument with QIAsymphony^®^ Midi Kit (Qiagen, Venlo, The Netherlands), according to the manufacturer’s instructions. DNA quality was assessed by 1% agarose gel electrophoresis, and concentration was measured using the Nanodrop ND 1000 spectrophotometer (NanoDrop Technologies Inc., Wilmington, DE, USA). All subjects were genotyped using a high-density Illumina SNP array (Illumina, Inc., San Diego, CA, USA). A list of SNPs within SweetG, compiled through a literature review (Table S1), was extracted and analyzed.

### 4.3. Statistical Analysis

Demographic and clinical features were reported as means with standard deviations (SDs) for continuous variables and as absolute frequencies and percentages for categorical variables. To explore the relationships among clinical features, a pairwise correlation matrix was constructed. Given the presence of both continuous and dichotomous variables, we applied both Pearson and Spearman correlation coefficients to ensure appropriate and reliable estimation of associations.

Genotypic and allelic frequencies for the candidate SNPs were reported as percentages. Allele frequencies in the study population were compared with those from gnomAD v4.1.0 Non-Finnish European reference datasets [37] using a binomial test.

All SNPs identified as statistically significant in the previous analysis were further examined for potential associations with the clinical features of PCD. Associations were evaluated using Fisher’s exact test or logistic regression models (adjusted for age and sex) for binary outcomes (*situs inversus*, neonatal respiratory distress, asthma, allergy, bronchiectasis, *Pseudomonas aeruginosa* colonization, CRS, nasal polyposis, respiratory exacerbations), and linear regression models (adjusted for age and sex) for continuous outcomes (Body Mass Index, modified Bhalla score, Bronchiectasis Severity Index, FEV_1_, SNOT-22 score).

A *p*-value of <0.05 was considered statistically significant. The statistical analyses were conducted using R software version 4.5.1 (www.r-project.org).

### 4.4. Ethical Statement

The study protocol was approved by the Ethics Committee of the Policlinico Hospital, Milan, Italy (protocol code No. 16 19 March 2007 and following revisions), and written informed consent was obtained from all adult participants or from the parents or legal guardians of participating children.

## Figures and Tables

**Table 1:** Demographic and clinical characteristics of PCD subjects.

	Patients n = 34
Male gender, n (%)	15 (44.1)
Age (years), mean (SD)	37.8 (16.9)
Age range	10.0, 69.0
BMI (kg/m^2^), mean (SD)	22.3 (2.8)
*Situs inversus*, n (%)	20 (58.8)
Neonatal respiratory distress, n (%)	7 (20.6)
Allergy, n (%)	9 (26.5)
Asthma, n (%)	7 (20.6)
Chronic rhinosinusitis, n (%)	23 (67.4)
Nasal polyposis, n (%)	16 (47.1)
SNOT-22 score, mean (SD)	32.3 (22.1)
Bronchiectasis, n (%)	24 (70.6)
Bronchiectasis Severity Index, mean (SD)	3.6 (3.3)
mBhalla, mean (SD)	13.1 (10.4)
Respiratory exacerbations ≥ 2/year, n (%)	22 (64.7)
PA colonization, n (%)	10 (29.4)
FEV_1_ (% of the predicted value), mean (SD)	81 (18.3)
n° lung lobes ≥ 2, n (%)	19 (59.4)

BMI: Body Mass Index; PA: *Pseudomonas aeruginosa* colonization; FEV_1_: Forced Expiratory Volume in 1 s.

**Table 2:** Significant results of the allele frequencies comparison among PCD patients and gnom-AD Non-Finnish European data of the selected list of SNPs.

SNP ID	Gene	Alleles REF/ALT	AF PCD	AF gnomAD-NFE	*p*-Value
rs838133	*FGF21*	A/G	0.6912	0.5687	0.0494
rs838145	*FGF21*	G/A	0.7206	0.5704	0.0138
rs2654185	*SLC2A4*	A/C	0.7794	0.6249	0.0082
rs5415	*SLC2A4*	T/C	0.8235	0.6928	0.0179

REF: reference allele; ALT: alternative allele; AF: allele frequency, referred to the alternative allele; NFE: Non-Finnish European populations; *p*-value: *p*-value from the binomial test.

## Data Availability

The original contributions presented in this study are included in the article/Supplementary Materials. Further inquiries can be directed to the corresponding author(s).

## Supplementary Materials

The supporting information can be downloaded at https://www.mdpi.com/article/10.3390/ijms27031234/s1.

## Abbreviations

The following abbreviations are used in this manuscript:

SNPs	Single-nucleotide polymorphisms
PCD	Primary ciliary dyskinesia
CRS	Chronic rhinosinusitis
BMI	Body mass index
LM	Lund–McKay score
BSI	Bronchiectasis severity index
ASL	Airway surface liquid
