# Supplementary material for: Association of Single-Nucleotide Polymorphisms in Sweet Taste Perception and Intake Genes with Primary Ciliary Dyskinesia and Its Clinical Phenotypes
Source: Int J Mol Sci. 2026 Jan 26;27(3):1234. doi: 10.3390/ijms27031234 (PMC12897696; doi:10.3390/ijms27031234)
Supplement: Supplementary file 1 [file ijms-27-01234-s001.zip › ijms-3993680-supplementary.pdf]

Table S1. Diagnostic findings in patients with a definitive diagnosis of PCD.

**HSVA**	
Immotile cilia	19 (55.9 %)
Abnormal beating	2 (5.9 %)
Normal beating	1 (2.9 %)
Inconclusive/not performed	12 (35.3 %)
**TEM Ultrastructure**	
Class 1 defects	16 (47 %)
Class 2 defects	9 (26.5 %)
Normal	4 (11.8 %)
Inconclusive/not performed	5 (14.7 %)
**Genetics**	
Positive	23 (67.7 %)
Negative	11 (32.3 %)

HSVA: high-speed video analysis; TEM: transmission electron microscopy

Table S2. List of the selected SNPs in sweet taste genes.

SNP id	Gene	Chromosome	Position	Alleles	Reference	Consequence
rs838145	*FGF21*	19	48745473	A/G	10.1093/ajcn/nqz043	Upstream gene variant
rs838133	*FGF21*	19	48756272	G/A	10.1093/ajcn/nqz043	Synonymous variant
rs11642841	*FTO*	16	53811575	C/A	10.1093/ajcn/nqz043	Intron variant
rs17457384	*GABRB2*	5	161274322	T/G	10.1093/ajcn/nqz043	Downstream gene variant
rs5400	*GLUT2*	3	171014511	G/A	10.1152/physiolgenomics.00148.2007	missense variant
rs17260734	*GNAT3*	7	80514039	T/A	10.3390/nu11071491	Upstream gene variant
rs7792845	*GNAT3*	7	80478482	G/A	10.3390/nu11071491	Upstream gene variant
rs6467192	*GNAT3*	7	80494683	T/C	10.1093/ajcn/nqz043	Intron variant
rs6975345	*GNAT3*	7	80522053	T/C	10.1093/ajcn/nqz043	Intron variant
rs2654185	*SLC2A4*	17	7280043	A/C	10.3390/nu11071491	Upstream gene variant
rs5415	*SLC2A4*	17	7281162	T/C	10.3390/nu11071491	Upstream gene variant
rs4908923	*TAS1R1*	1	6554475	G/A	10.3390/nu11071491	Upstream gene variant
rs9988418	*TAS1R2*	1	18839606	C/T	10.3390/nu17060949	Missense variant
rs28652778	*TAS1R2*	1	18868501	C/T	10.3390/nu11071491	Upstream gene variant
rs28374389	*TAS1R2*	1	18849352	T/C	10.3390/nu11071491	Missense variant
rs4920564	*TAS1R2*	1	18852610	G/T	10.1159/000430886	Intron variant
rs35424002	*TAS1R3*	1	1334606	G/A	10.1002/fsn3.2401	3 prime UTR variant

Table S3. Results of the allele frequencies comparison among PCD patients and gnom-AD Non-Finnish European data of the selected list of SNPs.

SNP id	Gene	Alleles REF/ALT	AF PCD	AF gnomAD-NFE	p-value
rs838133	*FGF21*	A/G	0.6912	0.5687	0.0494*
rs838145	*FGF21*	G/A	0.7206	0.5704	0.0138*
rs11642841	*FTO*	C/A	0.3676	0.4091	0.5386
rs17457384	*GABRB2*	T/G	0.0588	0.0455	0.553
rs5400	*GLUT2*	G/A	0.1765	0.1321	0.2811
rs17260734	*GNAT3*	T/A	0.4706	0.4776	1
rs6467192	*GNAT3*	G/A	0.1765	0.1204	0.1878
rs6975345	*GNAT3*	T/C	0.1765	0.1208	0.1886
rs7792845	*GNAT3*	T/C	0.5882	0.5907	1
rs2654185	*SLC2A4*	A/C	0.7794	0.6249	0.0082*
rs5415	*SLC2A4*	T/C	0.8235	0.6928	0.0179*
rs4908923	*TAS1R1*	G/A	0.8824	0.8718	1
rs9988418	*TAS1R2*	C/T	0.0147	0.0081	0.4231
rs28652778	*TAS1R2*	C/T	0.2647	0.233	0.5659
rs28374389	*TAS1R2*	T/C	0.2059	0.2121	1
rs4920564	*TAS1R2*	G/T	0.6324	0.6211	0.901
rs35424002	*TAS1R3*	G/A	0.0294	0.0372	1

PCD: Primary Ciliary Dyskinesia; REF: reference allele; ALT: alternative allele; AF: allele frequency, referred to the alternative allele; NFE: Non-Finnish European populations; p-value: p-value from binomial test. * indicates statistically significant values (<0.05).

Table S4. Results of regression models assessing the association between PCD clinical features and SNPs in the *SLC2A4* and *FGF21* genes.

Clinical Parameter	Gene	SNP	Model	Beta/OR	P-value
Allergy	*SLC2A4*	rs2654185	Logistic	1.621	0.5975
Asthma	*SLC2A4*	rs2654185	Logistic	1.891	0.5202
BMI	*SLC2A4*	rs2654185	Linear	0.08	0.9288
Bronchiectasis	*SLC2A4*	rs2654185	Logistic	0.56	0.55
Bronchiectasis severity index	*SLC2A4*	rs2654185	Linear	-0.07	0.9467
Chronic Rhinosinusitis	*SLC2A4*	rs2654185	Logistic	0.037	0.0607
FEV1	*SLC2A4*	rs2654185	Linear	1.66	0.7792
mBhalla	*SLC2A4*	rs2654185	Linear	-0.02	0.9963
n Lung Lobes ≥2	*SLC2A4*	rs2654185	Logistic	0.525	0.7052
Nasal Polyposis	*SLC2A4*	rs2654185	Logistic	0.831	0.8361
Neonatal Respiratory Distress	*SLC2A4*	rs2654185	Logistic	8.202	0.0809
PA Colonization	*SLC2A4*	rs2654185	Logistic	1.385	0.7259
Respiratory Exacerbations ≥2	*SLC2A4*	rs2654185	Logistic	0.438	0.3515
Situs Inversus	*SLC2A4*	rs2654185	Logistic	0.561	0.4647
SNOT-22 score	*SLC2A4*	rs2654185	Linear	-3.77	0.5996
Allergy	*SLC2A4*	rs5415	Logistic	0.824	0.8341
Asthma	*SLC2A4*	rs5415	Logistic	3.533	0.2978
BMI	*SLC2A4*	rs5415	Linear	0.25	0.778
Bronchiectasis	*SLC2A4*	rs5415	Logistic	0.547	0.5269
Bronchiectasis severity index	*SLC2A4*	rs5415	Linear	-0.63	0.5858
Chronic Rhinosinusitis	*SLC2A4*	rs5415	Logistic	0.005	0.0492*
FEV1	*SLC2A4*	rs5415	Linear	-3.35	0.5853
mBhalla	*SLC2A4*	rs5415	Linear	-0.74	0.8272
n Lung Lobes ≥2	*SLC2A4*	rs5415	Logistic	0.695	0.8015
Nasal Polyposis	*SLC2A4*	rs5415	Logistic	0.706	0.7037
Neonatal Respiratory Distress	*SLC2A4*	rs5415	Logistic	4.805	0.1861
PA Colonization	*SLC2A4*	rs5415	Logistic	0.673	0.6821
Respiratory Exacerbations ≥2	*SLC2A4*	rs5415	Logistic	0.846	0.8471
Situs Inversus	*SLC2A4*	rs5415	Logistic	1.262	0.7729

SNOT-22 score	*SLC2A4*	rs5415	Linear	-4.83	0.5162
Allergy	*FGF21*	rs838133	Logistic	0.345	0.2611
Asthma	*FGF21*	rs838133	Logistic	0.208	0.1937
BMI	*FGF21*	rs838133	Linear	-0.95	0.2686
Bronchiectasis	*FGF21*	rs838133	Logistic	1.174	0.861
Bronchiectasis severity index	*FGF21*	rs838133	Linear	-0.05	0.9664
Chronic Rhinosinusitis	*FGF21*	rs838133	Logistic	0.561	0.5393
FEV1	*FGF21*	rs838133	Linear	-7.1	0.2288
mBhalla	*FGF21*	rs838133	Linear	0.38	0.9071
n Lung Lobes ≥2	*FGF21*	rs838133	Logistic	0.207	0.3872
Nasal Polyposis	*FGF21*	rs838133	Logistic	3.701	0.1731
Neonatal Respiratory Distress	*FGF21*	rs838133	Logistic	0.454	0.4139
PA Colonization	*FGF21*	rs838133	Logistic	0.787	0.7906
Respiratory Exacerbations ≥2	*FGF21*	rs838133	Logistic	2.091	0.4084
Situs Inversus	*FGF21*	rs838133	Logistic	10.766	0.0145*
SNOT-22 score	*FGF21*	rs838133	Linear	11.08	0.1182
Allergy	*FGF21*	rs838145	Logistic	0.995	0.9948
Asthma	*FGF21*	rs838145	Logistic	0.957	0.9628
BMI	*FGF21*	rs838145	Linear	0.03	0.9755
Bronchiectasis	*FGF21*	rs838145	Logistic	0.634	0.6569
Bronchiectasis severity index	*FGF21*	rs838145	Linear	-1.18	0.2904
Chronic Rhinosinusitis	*FGF21*	rs838145	Logistic	3.429	0.268
FEV1	*FGF21*	rs838145	Linear	-3.29	0.5798
mBhalla	*FGF21*	rs838145	Linear	-5.38	0.0929
n Lung Lobes ≥2	*FGF21*	rs838145	Logistic	0.207	0.3202
Nasal Polyposis	*FGF21*	rs838145	Logistic	0.874	0.8822
Neonatal Respiratory Distress	*FGF21*	rs838145	Logistic	0.644	0.6349
PA Colonization	*FGF21*	rs838145	Logistic	0.422	0.3351
Respiratory Exacerbations ≥2	*FGF21*	rs838145	Logistic	0.761	0.766
Situs Inversus	*FGF21*	rs838145	Logistic	4.496	0.0643
SNOT-22 score	*FGF21*	rs838145	Linear	7.06	0.3246

BMI: Body Mass Index; PA: *Pseudomonas aeruginosa* colonization; FEV_1: Forced Expiratory Volume in 1 second. * indicates statistically significant values (<0.05).
